# Fermented and nonfermented soy foods and the risk of breast cancer in a Japanese population‐based cohort study

**DOI:** 10.1002/cam4.3677

**Published:** 2020-12-19

**Authors:** Ritsuko Shirabe, Eiko Saito, Norie Sawada, Junko Ishihara, Ribeka Takachi, Sarah Krull Abe, Taichi Shimazu, Taiki Yamaji, Atsushi Goto, Motoki Iwasaki, Manami Inoue, Shoichiro Tsugane, JPHC Study Group

**Affiliations:** ^1^ School of Public Health Graduate School of Medicine The University of Tokyo Bunkyo‐ku Japan; ^2^ Division of Cancer Statistics Integration Center for Cancer Control and Information Services National Cancer Center Chuo‐ku Japan; ^3^ Epidemiology and Prevention Group Center for Public Health Sciences National Cancer Center Chuo‐ku Japan; ^4^ Department of Food and Life Science School of Life and Environmental Science Azabu University Sagamihara Japan; ^5^ Department of Food Science and Nutrition Faculty of Human Life and Environment Nara Women's University Nara Japan

**Keywords:** breast cancer, fermented soy, Japan, prospective cohort study

## Abstract

**Background:**

Although preclinical studies suggest that fermented soy foods may have a protective effect against breast cancer, no prospective cohort studies have examined this association.

**Objective:**

Our study examined the association between fermented and nonfermented soy food intake and breast cancer risk using a population‐based prospective cohort study in Japan.

**Methods:**

We included a total of 47,614 women aged 45–74 years in an analysis of the Japan Public Health Center‐based Prospective Study (JPHC Study). A validated food frequency questionnaire (FFQ) was used for the assessment of dietary intake. Breast cancer incidence was analyzed by multivariate Cox proportional hazards regression models.

**Results:**

During an average of 15.5 years of follow‐up, 825 breast cancer cases were newly identified. We found no association of intake of soy foods with breast cancer risk, regardless of fermentation, with multivariate hazard ratios (HRs) and 95% confidence intervals (CIs) for the highest quartiles of fermented and nonfermented soy food intake of 0.94 (0.67, 1.32) and 1.15 (0.85, 1.57) compared with the lowest quartile (*p* for trend = 0.305 and 0.393). Unlike nonfermented soy, higher intake of fermented soy foods was associated with a significant decrease in the risk of nonlocalized breast cancer. The HR and 95% CI in the highest compared to lowest intake category of fermented soy foods was 0.53 (0.28, 0.99) versus nonfermented soy foods 0.85 (0.51, 1.42) (*p* for trend = 0.026 and 0.797).

**Conclusions:**

Our analyses showed that fermented soy foods had no association with overall breast cancer but may be associated with decreased risk of nonlocalized breast cancer.

## Graphical abstract

We investigated the association between fermented and non‐fermented soy food intake and breast cancer risk using a population‐based prospective cohort study in Japan. Our analyses showed that fermented soy foods had no association with overall breast cancer but may be associated with decreased risk of non‐localized breast cancer.

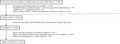

## 1 INTRODUCTION

Incidence rates of breast cancer vary among regions, with an age‐standardized incidence rate per 100,000 of 34.4 in Asia and 84.8 in Northern America in 2018.^1^ Although the incidence rate has been historically lower in Asia than in Western countries, it has nevertheless more than tripled in the last three decades in Japan, from 24.8 per 100,000 in 1985 to 82.6 per 100,000 in 2015. Among risk factors evaluated to date,^2^ this increase in incidence in Japan may be partly explained by increasing adoption of a Western‐style diet^4^, ^5^ and decreased physical activity,^5^ as well as changes in reproductive pattern.^6^ Other factors such as tobacco and alcohol use should not be neglected.^2^

Comparison of dietary patterns between Western and Asian countries reveals that Asians, including Japanese, have traditionally consumed relatively high amounts of soy foods.^7^ Soybeans are a rich source of bioactive isoflavones. Isoflavones are a group of phytoestrogens which have similar structures to the estrogens, one of the ovarian hormones. The endogenous estrogens appear likely to have a positive association with the risk of breast cancer.^9^, ^10^ Phytoestrogens act like estrogens in mammals and may weaken endogenous estrogens by binding the estrogen receptor.^11^, ^12^ Human beings consume soy in various ways, broadly divided into fermented and nonfermented status. In the human body, isoflavones in fermented soy foods are absorbed more easily and quickly than those in nonfermented soy foods.^13^, ^14^

A plethora of epidemiological studies exists on the association between soy consumption and breast cancer. However the evidence is inconsistent. Two meta‐analyses^13^, ^14^ showed a weak but statistically significant inverse association between soy consumption and risk of breast cancer. These studies warrant careful interpretation, however, because most prospective cohort studies^15^, ^16^, ^17^, ^18^, ^19^, ^20^ found no association between soy foods or genistein intake and risk of breast cancer. In addition, previous studies showed different results among countries. One review^20^ found that Asian people showed a significantly decreased trend in breast cancer risk with increased soy intake, whereas Western studies found no association. With regard to fermented soy foods, one case‐control study indicated that the risk of breast cancer decreased among women with high intake of both soy isoflavone and dairy products containing bacteria.^21^ Our previous cohort analysis^22^ showed an inverse association with breast cancer risk by miso soup (fermented soybean paste soup) intake. Nevertheless, no cohort study has evaluated possible differences in the effects of fermented and nonfermented soy foods.

To assess these different effects, the association of fermented and nonfermented soy foods should be evaluated separately. In this study, we examined the association between fermented and nonfermented soy intake and the risk of breast cancer incidence in a prospective cohort study in Japan.

## 2 SUBJECTS AND METHODS

### 2.1 Study population

**FIGURE 1:**
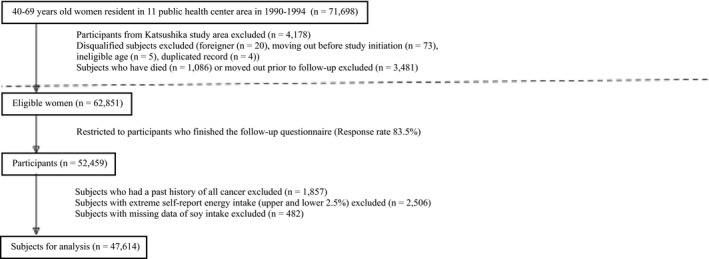
Participants flow chart

This study was a prospective cohort study conducted based on the Japan Public Health Center‐based Prospective Study (JPHC study), which targeted 140,420 residents (68,722 men and 71,698 women) to investigate the associations between lifestyle and illness such as cardiovascular disease and cancer. The JPHC study is being conducted in 11 public health centers (PHCs) across Japan. Cohort I was established in 1990, and enrolled residents aged 40–59 years who had registered their addresses with the Akita (Yokote), Iwate (Ninohe), Nagano (Saku), Tokyo (Katsushika), and Okinawa (Ishikawa) local governments at the time of baseline collection. Cohort II was established in 1993, and enrolled residents aged 40–69 and who had registered their addresses with the Niigata (Kashiwazaki), Ibaraki (Mito), Osaka (Suita), Kochi (Chuohigashi), Nagasaki (Kamigoto), and Okinawa (Miyako) local areas at the time of baseline collection. The JPHC Study been approved by the Institutional Review Board of the National Cancer Center (approval number 2001‐021). Completion of the questionnaire after receiving a briefing about the study purposes and methods was considered informed consent. Details of the JPHC Study have been previously published.^23^ In this study, we included women only. Subjects from the Katsushika, Tokyo PHC area were excluded (n = 4,178) as cancer incidence data were not accessible. We also excluded non‐Japanese subjects (n = 20), late report of emigration before the start of the follow‐up (n = 73), incorrect birth date (n = 5), or duplicate registration (n = 2). In 1995–1999, subjects answered a self‐administered 5‐year follow‐up questionnaire, consisting of comprehensive information on demographic characteristics, anthropometric measurements, medical histories, dietary intake, smoking and drinking habits, menopausal status and exogenous female hormone use, physical activity and the other lifestyle habits. This information, based on the 5‐year follow‐up questionnaire, was used as the starting point of this study. Subjects who had died (n = 1,086) or moved out (n = 3,481) prior to follow‐up were excluded. There were 39 (3.6%) breast cancer cases among the 1,086 deaths prior to follow‐up. This mortality rate did not differ from the national data of Japan in the study period.^24^ Of the 62,851 women who were eligible for participation, 52,459 answered the questionnaire, giving a response rate of 83.5%. Women with a past history of any cancer (n = 1,857), extreme energy intake (upper and lower 2.5%, n = 2,506), or missing data on dietary soy intake (n = 482) were excluded. Finally, a total of 47,614 women, aged 45–74 years at the start of this study were included in this analysis (Figure 1).

### 2.2 Exposure

Dietary intake was assessed with a validated self‐administered food frequency questionnaire (FFQ) administered at the time of the 5‐year follow‐up survey. Intake frequency of 138 foods and beverages during the previous one year using standard portion sizes was collected.^25^ The Standard Tables of Food Composition (5th revised edition) in Japan was used to calculate food intake per day. Within the questionnaire, subjects were asked how often they consumed individual soy products “miso soup” (fermented soybean paste soup), “tofu (in miso soup)”, “tofu (for other dishes like boiled tofu and cold tofu)”, “yushidofu” (predrained tofu), “koyadofu and shimitofu” (dried tofu), “aburaage” (fried tofu), “natto” (fermented soybean), and “soymilk”. Miso soup consumption was divided into six frequency categories: almost never, 1–3 times/month (m), 1–2 times/week (w), 3–4 times/w, 5–6 times/w, and daily. The participants were then asked about their daily consumption of miso soup bowls in the nine categories of <1, 1, 2, 3, 4, 5, 6, 7–9, and ≥10. The nine frequency categories of the other soy foods consumed were never, 1–3 times/m, 1–2 times/w, 3–4 times/w, 5–6 times/w, once a day (d), 2–3 times/d, 4–6 times/d, and ≥7 times/d. The consumption of soymilk was divided into the nine categories of never, 1–2 times /w, 3–4 times/w, 5–6 times/w, 1 glass/d, 2–3 glasses/d, 4–6 glasses/d, 7–9 glasses/d, and ≥10 glasses/d. Standard portion sizes were specified as 150 ml (one bowl of miso soup), 20 g (tofu for miso soup), 75 g (tofu for other dishes), 150 g (predrained tofu), 60 g (dried tofu), 2 g (fried tofu), and 50 g (natto), and 200 ml (soymilk). These standard portion sizes were stated and participants chose portion sizes relative to them as small (less than half the specified size), same and large (more than 1.5 times). In this study, frequency of intake was multiplied by standard portion and relative portion size, where available, for each item in the FFQ to obtain the daily intake of individual food items. To newly assess the different effects of fermented and nonfermented soy foods, we categorized miso and natto as “fermented soy” and tofu, dried tofu, fried tofu and soymilk as “non‐fermented soy”. In our previous validation study of isoflavone intake in this population,^26^ only three soy foods (miso, natto and tofu) contributed to more than 80% of total isoflavone intake in FFQ estimates. The Spearman rank correlation indicated high correlation of the main isoflavones (genistein and daidzein) (coefficient was 0.621 in this study). Therefore, we used genistein intake as representative of “isoflavone” intake throughout this article. Genistein intake was calculated with a food composition table specifically developed for the FFQ of isoflavones in Japanese foods.^28^, ^29^

The validity of food item intake assessed with the FFQ was evaluated for subsamples of 498 subjects using 14‐ or 28‐day dietary records and the FFQ used in the 5‐year follow‐up survey.^29^ The validity and reproducibility of genistein intake assessed from the FFQ was also evaluated from subsamples of 247 subjects.^26^ The Spearman rank correlation coefficient between energy‐adjusted soy products intake (g/d) estimated from dietary records and from the FFQ for women was 0.33.^29^ Spearman's correlation coefficients for energy‐adjusted genistein intake (mg/d) from dietary records and the FFQ were 0.65 (cohort I) one year apart, showing correlation coefficients for energy‐adjusted intake of soy products in women of 0.56,^29^ whereas that for energy‐adjusted genistein were 0.61 (cohort I)^30^ and 0.41 (cohort II).^31^ The validity and reproducibility of the FFQ were considered sufficient for the present analyses.

### 2.3 Outcome

Breast cancer incidence was defined as the primary outcome of this study. Cases were identified by active patient notification with data coming from major local hospitals and linked with data from population‐based cancer registries. Use of data was approved by local governments responsible for the registries. Breast cancer cases were classified using codes C500‐C509 defined in the Third Edition of the International Classification of Diseases for Oncology. We categorized breast cancer with metastasis to regional lymph nodes, or adjacent or distant organs as nonlocalized cancer. We did not include cancer in situ as cases. Death certificates provided supplementary information. A total of 879 breast cancer cases were identified. Among these, 19 cases depended on death certificate notification (DCN = 2.2%), for which diagnostic information was unavailable for 18 cases (DCO = 2.1%). For 805 (96.3%) cases, diagnosis was microscopically verified. Only the first incidence of breast cancer was considered in this analysis in women with more than one diagnosis.

### 2.4 Follow‐up

Follow‐up started with the 5‐year follow‐up survey administration date. Person years of follow‐up were accumulated until the date of cancer diagnosis, death, migration out of a study area, or end of follow‐up (December 31, 2013), whichever occurred first. Residential registers of each municipality confirmed residence status and survival once a year in the study areas and the municipal office of the area the subjects moved to. Dates of death were verified by linkage with death registrations under the Ministry of Health, Labor, and Welfare.

### 2.5 Statistical analysis

For the primary outcome, we examined the association between soy food intake and breast cancer incidence. Intake of soy foods and isoflavone was energy‐adjusted using the residual approach.^32^ Participants were subdivided into quartiles based on soy food intake (total, fermented, nonfermented and each food item except soymilk and dried tofu) and isoflavone (total, fermented and nonfermented). For soymilk and dried tofu, we divided participants into two groups (consume some or none) because many participants consumed none (91% for soymilk and 48% for dried tofu). We used a raw model (only adjusted for age and area) and hazard ratios (HRs) and 95% confidence intervals (CIs) were estimated using multivariable Cox proportional hazards regression models, considering the lowest category as reference. Tests for linear trend were estimated with the median value of each food and isoflavone category. Multivariable Cox proportional hazards models included age (continuous), area (10 public health centers), body mass index (BMI) (continuous), height (continuous), smoking status (never, former or current), cigarettes number/day (none, 1–20, 21–30, ≥30), alcohol intake (never, nonregular drinker <150 g of ethanol/w, regular drinker ≥150 g of ethanol/w), total energy intake (continuous), physical activity (Metabolic equivalents [METs]) (continuous), past history of diabetes (yes/no), family history (1st degree‐relative) of breast cancer (yes/no), received mammography during the previous year (yes/no), age at menarche (continuous), age at first birth (continuous), history of breastfeeding (yes/no), number of deliveries (continuous), menopausal age (continuous), and use of exogenous female hormones, including oral contraceptives and menopausal hormone treatment (never, ever). Physical activity of each participant was calculated by daily time spent on hard work or sports, sitting and walking or standing. Age at first birth and age at menopause cut‐offs were based on the distribution of the Japanese population in the study's generation and regarded outliers as missing values. For women whose menopausal status was not available, we used 51 years as a cut‐off for menopausal age at the time of 5‐year follow‐up, based on data showing that approximately half of Japanese women had become postmenopausal by that age.^33^ The number of missing values for each variable is reported in Table 1. The distribution of breast cancer cases did not differ between individuals with complete and incomplete data (1.8% vs. 1.9%), and missing data were therefore considered to be missing at random. We assumed multivariate normality and used multiple imputation. Twenty imputed datasets were created for the number of deliveries, including variables of BMI, height, smoking status, number of cigarette smoked per day, alcohol intake, total energy intake, past history of diabetes, age at menarche, age at first birth, history of breastfeeding, menopausal age, and use of exogenous female hormones. Regression analyses were performed 20 times and their results were combined in Stata statistical software. We performed a sensitivity analysis which excluded cases diagnosed within 5 years to exclude participants who could have had breast cancer but were not diagnosed at the start of follow‐up, as well as an analysis which included only those cases detected by subjective symptoms to eliminate possible bias by subjects who consumed high soy foods and received mammography (Table 1).

**TABLE 1:** Subject characteristics according to quartile of energy‐adjusted total soy intake among Japanese women in the JPHC Study (n = 47,614)

	Q1 low (n = 11,904)	Q2 (n = 11,903)	Q3 (n = 11,904)	Q4 high (n = 11,903)	*p* value	Number of missing value
Soy foods (g/day)	30.1 ± 10.4	56.4 ± 6.8	83.3 ± 9.4	169 ± 107	<0.001	
Fermented	12.9 ± 8.8	25.4 ± 12.8	35.8 ± 17.9	44.8 ± 31.5	<0.001	
Miso	8.6 ± 7.3	15.0 ± 9.9	18.4 ± 11.6	20.2 ± 13.1	<0.001	
Natto	4.5 ± 7.0	10.3 ± 12.3	17.7 ± 19.6	27.6 ± 39.3	<0.001	
Non‐fermented	17.2 ± 9.6	30.9 ± 13.4	47.4 ± 19.3	126 ± 113	<0.001	
Tofu	14.8 ± 8.8	26.4 ± 12.9	39.0 ± 19.0	84.2 ± 71.8	<0.001	
Soymilk	0.1 ± 1.5	0.6 ± 4.6	2.9 ± 10.7	30.0 ± 99.1	<0.001	
Fried tofu	0.3 ± 0.4	0.5 ± 0.6	0.6 ± 0.8	0.7 ± 1.2	<0.001	
Dried tofu	2.2 ± 3.9	3.5 ± 5.7	5.2 ± 9.3	11.4 ± 28.3	<0.001	
Isoflavone (mg/day)	9.1 ± 3.9	17.8 ± 4.7	26.4 ± 7.1	44.8 ± 23.1	<0.001	
Fermented	5.1 ± 3.8	10.7 ± 6.2	15.9 ± 9.3	20.8 ± 17.8	<0.001	
Nonfermented	4.0 ± 2.3	7.1 ± 3.2	10.5 ± 4.4	24.2 ± 19.3	<0.001	
Age (years)	56.5 ± 8.5	57.0 ± 8.0	57.2 ± 7.7	58.3 ± 7.5	<0.001	
BMI	23.3 ± 3.2	23.4 ± 3.2	23.5 ± 3.1	23.8 ± 3.2	<0.001	1,274 (2.7%)
Height (cm)	152 ± 5.6	152 ± 5.5	152 ± 5.6	151 ± 5.6	<0.001	5,604 (11.8%)
Current smoker (yes, %)	8.5	5.5	4.7	4.6	<0.001	2,932 (6.1%)
Regular drinker (yes, %)	22.4	20.1	18	14.9	<0.001	1,377 (2.9%)
Past history of diabetes (yes, %)	3.8	3.9	3.8	5.5	<0.001	
Family History (yes, %)	0.6	0.4	0.5	0.3	0.018	
Mammography (yes, %)	8.4	9.4	9.7	12.2	<0.001	
Physical activity (METs/day)	31.8 ± 5.8	31.9 ± 5.8	32.1 ± 5.8	31.9 ± 5.8	<0.001	1,593 (3.3%)
Postmenopausal (yes, %)	71.6	76.5	78.6	83.3	<0.001	2,927 (6.1%)
Hormone therapy (yes, %)	2.7	2.5	2.8	2.8	0.268	2,698 (5.7%)
Age at menarche (years)	14.4 ± 1.9	14.6 ± 1.8	14.7 ± 1.8	15.1 ± 2.0	<0.001	6,501 (13.7%)
Age at first birth (years)	25.0 ± 3.5	25.0 ± 3.3	24.9 ± 3.3	24.6 ± 3.4	<0.001	10,456 (22%)
Number of deliveries	2.5 ± 1.3	2.6 ± 1.2	2.6 ± 1.2	2.8 ± 1.4	<0.001	8,891 (18.7%)
Breast fed (yes, %)	85.8	87.1	87.5	88.1	<0.001	9,873 (20.7%)
Energy intake (kcal/day)	1860 ± 584	1880 ± 558	1870 ± 545	1810 ± 838	<0.001	

Note

Values are mean ± standard deviation unless otherwise indicated.

Abbreviations: %, percent; g, gram; kcal, kilocalorie;METs, Metabolic equivalents; mg, milligram; Q, quartile.

Further, we analyzed the association by cancer extent (localized or nonlocalized) to evaluate the effect of fermented soy foods. Finally, we also looked for interactions with menopausal status (premenopausal or postmenopausal) and BMI category (<23, 23–24.9, 25–26.9, ≥27). In the analyses of cancer extent and menopausal status, we conducted log‐rank tests to compare the results of two groups.

All tests were two‐sided and the threshold for significance was *p* < 0.05. All statistical analyses were conducted with Stata MP 14.

## 3 RESULTS

During an average of 15.5 years (interquartile range: 15.1–18.8) of follow‐up, 47,614 women contributed 738,504 person years, and 825 cases of breast cancer were newly identified. Mean age at diagnosis was 65.0 years (standard deviation = 8.7). Table 1 shows baseline characteristics of the study participants according to quartile of energy‐adjusted total soy food intake (the same number of subjects were equally distributed into the four quartiles). Total soy food intake ranged from a mean of 30.1 g/day in the lowest quartile to 169.2 g/day in the highest. Women who consumed more soy foods were more likely to be older, shorter, have received mammography, and menopausal; and less likely to be current smokers and regular drinkers.

Table 2 presents HRs and 95% CIs for breast cancer incidence according to quartile of soy foods and isoflavone intake in a raw model and in a multivariable‐adjusted model. Unlike the results of the raw model, no association was found for risk of breast cancer and soy food and isoflavone intake, regardless of fermented or nonfermented status. No association was observed even after exclusion of incident cases during the first 5 years and limiting analysis to cases detected by subjective symptoms (data not shown).

**TABLE 2:** Hazard ratios (HRs) and 95% confidence intervals (CIs) for breast cancer according to quartile of soy foods and isoflavone intake among Japanese women of the JPHC Study

	Median (g/day)	No. of cases	Person years of follow‐up	HR1 (95% CI)	HR2 (95% CI)
Soy foods					
Total					
Q1	31.8	208	179,227.19	1	1
Q2	56.4	218	184,154.31	0.99 (0.82, 1.21)	1.06 (0.79, 1.43)
Q3	82.5	202	187,696.78	0.89 (0.73, 1.09)	0.99 (0.73, 1.34)
Q4	137	197	187,425.22	0.88 (0.71, 1.08)	1.07 (0.79, 1.46)
*p* trend				0.158	0.743
Fermented					
Q1	7.3	197	176,713.47	1	1
Q2	19.3	224	184,295.03	1.06 (0.87, 1.30)	1.25 (0.93, 1.69)
Q3	32.6	224	188,418.63	0.99 (0.80, 1.22)	1.02 (0.74, 1.41)
Q4	53.2	180	189,076.38	0.78 (0.62, 0.98)	0.94 (0.67, 1.32)
*p* trend				0.009	0.305
Miso					
Q1	2.9	219	175,358.09	1	1
Q2	9.8	209	181,581.91	0.93 (0.76, 1.12)	0.98 (0.73, 1.32)
Q3	17.7	216	189,349.77	0.89 (0.73, 1.09)	1.03 (0.76, 1.40)
Q4	29.1	181	192,213.74	0.72 (0.58, 0.89)	0.88 (0.64, 1.22)
*p* trend				0.002	0.458
Natto					
Q1	0.01	166	178,321.31	1	1
Q2	3.8	239	188,109.86	1.27 (1.02, 1.57)	1.42 (1.03, 1.97)
Q3	11.9	220	187,377.49	1.17 (0.94, 1.47)	1.26 (0.90, 1.77)
Q4	32.7	200	184,694.85	1.08 (0.85, 1.36)	1.08 (0.76, 1.54)
*p* trend				0.498	0.420
Non‐fermented					
Q1	13.5	198	181,425.87	1	1
Q2	28.2	218	185,100.36	1.07 (0.88, 1.30)	1.06 (0.79, 1.44)
Q3	47.7	209	186,354.53	1.02 (0.84, 1.24)	1.18 (0.88, 1.58)
Q4	98.5	200	185,622.75	0.99 (0.81, 1.22)	1.15 (0.85, 1.57)
*p* trend				0.714	0.393
Tofu					
Q1	10.3	197	183,180.53	1	1
Q2	22.1	211	185,034.35	1.05 (0.86, 1.28)	0.98 (0.73, 1.33)
Q3	37.3	230	185,361.45	1.13 (0.93, 1.37)	1.23 (0.93, 1.65)
Q4	74.1	187	184,927.18	0.92 (0.74, 1.13)	1.02 (0.75, 1.38)
*p* trend				0.287	0.867
Fried tofu					
Q1	0.02	207	185,318.46	1	1
Q2	0.2	203	184,644.98	0.99 (0.80, 1.21)	0.73 (0.53, 0.99)
Q3	0.4	199	183,903.99	0.95 (0.77, 1.18)	0.86 (0.63, 1.17)
Q4	1	216	184,636.08	1.01 (0.81, 1.26)	0.86 (0.63, 1.18)
*p* trend				0.843	0.963
Soymilk					
No	0	742	670,605.12	1	1
Yes	90.3	83	67,898.384	1.14 (0.90, 1.44)	1.34 (0.97, 1.86)
*p* value				0.264	0.078
Dried tofu					
No	0	390	344,672.51	1	1
Yes	10.7	435	393,831	1.01 (0.85, 1.19)	0.92 (0.72, 1.17)
*p* value				0.946	0.481
Isoflavone (mg/day)					
Total					
Q1	8.95	190	179,937.96	1	1
Q2	16.7	224	184,752.06	1.11 (0.92, 1.36)	1.29 (0.95, 1.75)
Q3	25.4	216	186,846.25	1.05 (0.85, 1.29)	1.18 (0.86, 1.61)
Q4	41.1	195	186,967.24	0.94 (0.76, 1.17)	1.10 (0.80, 1.52)
*p* trend				0.325	0.980
Fermented					
Q1	2.7	196	177,302.81	1	1
Q2	7.45	196	185,351.87	0.93 (0.76, 1.14)	0.95 (0.70, 1.29)
Q3	13.5	243	188,416.95	1.10 (0.89, 1.36)	1.05 (0.77, 1.43)
Q4	24.9	190	187,431.88	0.85 (0.68, 1.07)	0.87 (0.63, 1.22)
*p* trend				0.182	0.412
Nonfermented					
Q1	3.1	204	182,630.81	1	1
Q2	6.4	206	185,845.04	0.98 (0.81, 1.19)	1.04 (0.77, 1.40)
Q3	10.6	216	185,320.28	1.04 (0.85, 1.26)	1.12 (0.84, 1.51)
Q4	20.4	199	184,707.37	0.97 (0.79, 1.18)	1.19 (0.88, 1.60)
*p* trend				0.811	0.222

Note

HR1: Adjusted for age and area; HR2 Additionally adjusted for smoking status, number of cigarettes smoked per day, alcohol consumption, BMI, height, energy intake, physical activity, past history of diabetes, family history, received mammography, age at menarche, age at first birth, number of deliveries, menopausal age, hormone use, and breast feeding. The threshold for significance was *p* < 0.05.

Abbreviations: g, gram; mg, milligram; No, number; Q, quartile.

Information on cancer extent was available for 732 cases (89% of all). Table 3 shows the results of analysis by cancer extent. The HRs of nonlocalized cancer were statistically higher than those of localized cancer in this cohort (χ^2^ = 15.1, *p* = 0.0001). There was no association between either fermented and nonfermented soy foods and isoflavone intake and localized breast cancer. In contrast, nonlocalized breast cancer risk significantly decreased in the higher quartiles of fermented soy foods consumption compared with nonfermented. We reanalyzed the degree of cancer extent by menopausal status, but the results showed no meaningful change.

**TABLE 3:** Hazard ratios (HRs) and 95% confidence intervals (CIs) for breast cancer according to quartile of soy foods and isoflavone intake by cancer extent among Japanese women in the JPHC Study

	Median (g/day)	Localized cancer (n = 445)		Nonlocalized cancer (n = 287)	
		No. of cases	HR (95% CI)	No. of cases	HR (95% CI)
Soy foods					
Total					
Q1	31.8	106	1	77	1
Q2	56.4	120	1.20 (0.80, 1.79)	75	0.90 (0.54, 1.51)
Q3	82.5	115	1.26 (0.85, 1.88)	69	0.78 (0.46, 1.34)
Q4	137	104	1.19 (0.79, 1.80)	66	0.84 (0.49, 1.44)
*p* trend			0.545		0.555
Fermented					
Q1	7.3	113	1	62	1
Q2	19.3	123	1.28 (0.87, 1.89)	78	1.01 (0.59, 1.73)
Q3	32.6	111	0.93 (0.61, 1.43)	83	1.03 (0.60, 1.78)
Q4	53.2	98	1.17 (0.76, 1.81)	64	0.53 (0.28, 0.99)
*p* trend			0.793		0.026
Miso					
Q1	2.9	117	1	77	1
Q2	9.8	118	1.14 (0.77, 1.69)	69	0.72 (0.43, 1.21)
Q3	17.7	112	1.19 (0.79, 1.77)	79	0.71 (0.42, 1.19)
Q4	29.1	98	1.16 (0.76, 1.78)	62	0.56 (0.32, 0.98)
*p* trend			0.541		0.060
Natto					
Q1	0.01	84	1	62	1
Q2	3.8	138	1.64 (1.07, 2.50)	77	0.95 (0.53, 1.71)
Q3	11.9	113	1.18 (0.76, 1.88)	80	1.14 (0.64, 2.03)
Q4	32.7	110	1.21 (0.77, 1.92)	68	0.79 (0.43, 1.48)
*p* trend			0.709		0.356
Nonfermented					
Q1	13.5	91	1	83	1
Q2	28.2	123	1.57 (1.03, 2.39)	74	0.76 (0.46, 1.26)
Q3	47.7	124	1.81 (1.20, 2.72)	64	0.71 (0.43, 1.19)
Q4	98.5	107	1.52 (0.98, 2.35)	66	0.85 (0.51, 1.42)
*p* trend			0.268		0.797
Tofu					
Q1	10.3	98	1	74	1
Q2	22.1	118	1.20 (0.80, 1.80)	71	0.88 (0.53, 1.48)
Q3	37.3	127	1.54 (1.05, 2.28)	76	0.97 (0.58, 1.61)
Q4	74.1	102	1.21 (0.80, 1.84)	66	0.91 (0.53, 1.54)
*p* trend			0.548		0.824
Fried tofu					
Q1	0.02	111	1	73	1
Q2	0.2	95	0.64 (0.42, 0.98)	84	1.00 (0.60, 1.67)
Q3	0.4	118	1.03 (0.69, 1.54)	56	0.63 (0.35, 1.13)
Q4	1	121	0.96 (0.63, 1.47)	74	0.74 (0.42, 1.31)
*p* trend			0.457		0.255
Soymilk					
No	0	399	1	260	1
Yes	90.3	46	1.21 (0.79, 1.86)	27	1.62 (0.93, 2.80)
*p* value			0.376		0.087
Dried tofu					
No	0	207	1	148	1
Yes	10.7	238	1.08 (0.79, 1.48)	139	0.57 (0.37, 0.89)
*p* value			0.639		0.013
Isoflavone (mg/day)					
Total					
Q1	8.95	101	1	69	1
Q2	16.7	132	1.43 (0.96, 2.12)	66	1.07 (0.62, 1.84)
Q3	25.4	106	1.22 (0.80, 1.84)	83	1.15 (0.67, 1.97)
Q4	41.1	106	1.21 (0.80, 1.84)	69	0.90 (0.51, 1.59)
*p* trend			0.799		0.589
Fermented					
Q1	2.7	105	1	68	1
Q2	7.45	116	1.21 (0.82, 1.79)	61	0.46 (0.26, 0.83)
Q3	13.5	127	1.04 (0.68, 1.59)	84	0.81 (0.48, 1.37)
Q4	24.9	97	1.02 (0.66, 1.58)	74	0.57 (0.32, 1.02)
*p* trend			0.734		0.266
Nonfermented					
Q1	3.1	95	1	84	1
Q2	6.4	128	1.64 (1.09, 2.46)	60	0.58 (0.34, 1.00)
Q3	10.6	114	1.54 (1.02, 2.33)	73	0.78 (0.47, 1.28)
Q4	20.4	108	1.50 (0.98, 2.28)	70	1.00 (0.61, 1.61)
*p* trend			0.296		0.420

Note

HR: Adjusted for age, area, smoking status, number of cigarettes smoked per day, alcohol consumption, BMI, height, energy intake, physical activity, past history of diabetes, family history, received mammography, age at menarche, age at first birth, number of deliveries, menopausal age, hormone use and breast. The threshold for significance was *p* < 0.05.

Abbreviations: g, gram; mg, milligram; No, number; Q, quartile.

Table 4 shows the results of sub‐group analysis for breast cancer risk and soy food/isoflavone intake by menopausal status. The HRs of premenopausal women were statistically higher than those of postmenopausal women (χ^2^ = 4.56, *p* = 0.033). We did not observe a statistically significant tendency of breast cancer risk reduction by higher intake of fermented soy foods in either premenopausal or postmenopausal women.

**TABLE 4:** Hazard ratios (HRs) and 95% confidence intervals (CIs) for breast cancer according to quartile of soy foods and isoflavone intake by menopausal status among Japanese women in the JPHC Study

	Premenopausal women (n = 10,708/217 cases)				Postmenopausal women (n = 36,906/608 cases)			
	No. of cases	Median (g/day)	Person years of follow‐up	HR (95% CI)	No. of cases	Median (g/day)	Person years of follow‐up	HR (95% CI)
Soy foods								
Total								
Q1	73	31.2	52,061.10	1	135	32.1	127,166.08	1
Q2	66	56.4	44,495.73	1.04 (0.70, 1.56)	152	56.4	139,658.58	1.08 (0.80, 1.45)
Q3	45	82.1	41,300.75	0.77 (0.49, 1.22)	157	82.6	146,396.02	1.01 (0.75, 1.37)
Q4	33	135	32,334.35	0.71 (0.43, 1.19)	164	138	155,090.88	1.10 (0.81, 1.49)
*p* trend				0.119				0.602
Fermented								
Q1	55	7.48	46,283.49	1	142	7.30	130,366.82	1
Q2	62	19.0	45,630.18	1.14 (0.73, 1.77)	162	19.4	138,664.85	1.26 (0.94, 1.70)
Q3	66	32.1	42,981.10	1.19 (0.75, 1.90)	158	32.7	145,430.21	1.08 (0.79, 1.48)
Q4	34	52.0	35,210.43	0.73 (0.41, 1.29)	146	53.5	153,849.68	0.96 (0.69, 1.35)
*p* trend				0.274				0.386
Miso								
Q1	61	2.91	47,995.73	1	158	2.81	127,362.35	1
Q2	62	9.78	43,629.39	0.98 (0.65, 1.49)	147	9.85	137,952.52	0.95 (0.71, 1.27)
Q3	57	17.8	41,385.71	0.81 (0.52, 1.28)	159	17.7	147,964.07	0.98 (0.73,1.32)
Q4	37	28.4	37,181.11	0.67 (0.41, 1.12)	144	29.4	155,032.62	0.85 (0.62, 1.16)
*p* trend				0.093				0.314
Natto								
Q1	39	0.01	34,012.25	1	127	0.01	144,309.06	1
Q2	64	3.81	52,675.52	1.10 (0.66, 1.83)	175	3.78	135,434.34	1.45 (1.05, 2.01)
Q3	68	11.8	46,823.44	1.33 (0.80, 2.23)	152	11.9	140,554.05	1.30 (0.93, 1.82)
Q4	46	31.2	36,680.74	0.95 (0.53, 1.72)	154	33.1	148,014.11	1.19 (0.84, 1.67)
*p* trend				0.612				0.786
Nonfermented								
Q1	72	13.5	49,476.91	1	126	13.5	131,948.96	1
Q2	63	27.8	44,863.70	1.09 (0.72, 1.64)	155	28.4	140,236.66	1.10 (0.82, 1.48)
Q3	48	47.1	41,952.72	0.89 (0.57, 1.39)	161	47.7	144,,401.81	1.21 (0.91, 1.62)
Q4	34	93.9	33,898.61	0.87 (0.53, 1.43)	166	99.6	151,724.14	1.21 (0.90, 1.64)
*p* trend				0.448				0.250
Tofu								
Q1	64	10.4	48,467.26	1	133	10.3	134,713.28	1
Q2	62	21.9	44,906.36	1.15 (0.75, 1.76)	149	22.2	140,127.98	1.03 (0.76, 1.38)
Q3	56	37.2	41,092.30	1.14 (0.73, 1.78)	174	37.3	144,269.15	1.27 (0.96, 1.69)
Q4	35	72.1	35,726.03	0.91 (0.55, 1.53)	152	74.7	149,201.15	1.08 (0.80, 1.46)
*p* trend				0.618				0.624
Fried tofu								
Q1	49	0.02	40,037.54	1	158	0.02	145,280.92	1
Q2	67	0.18	45,624.78	1.17 (0.72, 1.90)	136	0.18	139,020.2	0.75 (0.55, 1.02)
Q3	52	0.42	46,798.60	1.00 (0.59, 1.71)	147	0.42	137,105.38	0.89 (0.65, 1.20)
Q4	49	1.04	37,731.02	1.17 (0.67, 2.04)	167	1.04	146,905.06	0.93 (0.68, 1.27)
*p* trend				0.724				0.671
Soymilk								
No	205	0.01	156,422.55	1	537	0.01	514,182.57	1
Yes	12	43.6	13,769.39	0.76 (0.39, 1.48)	71	47.3	54,128.995	1.32 (0.96, 1.82)
*p* value				0.425				0.090
Dried tofu								
No	112	0.01	81,726.54	1	278	0.01	262,945.97	1
Yes	105	4.88	88,465.40	0.91 (0.62, 1.34)	330	5.55	305,365.59	0.94 (0.74, 1.20)
*p* value				0.642				0.617
Isoflavone (mg/day)								
Total								
Q1	66	8.69	49,586.50	1	124	9.04	130,351.46	1
Q2	62	16.6	46,455.91	1.16 (0.76, 1.75)	162	16.8	138,296.14	1.32 (0.98, 1.79)
Q3	50	25.2	40,421.60	0.93 (0.58, 1.48)	166	25.4	146,424.64	1.21 (0.89, 1.65)
Q4	39	40.2	33,727.92	0.88 (0.53, 1.46)	156	41.4	153,239.31	1.16 (0.85, 1.60)
*p* trend				0.438				0.788
Fermented								
Q1	50	2.73	44,440.22	1	146	2.63	132,862.58	1
Q2	55	7.44	46,018.37	1.08 (0.67, 1.73)	141	7.46	139,333.5	0.95 (0.70, 1.29)
Q3	71	13.4	43,963.02	1.59 (0.99, 2.56)	172	13.6	144,453.93	1.11 (0.82, 1.51)
Q4	41	24.2	35,770.33	1.04 (0.59, 1.83)	149	25.0	151,661.55	0.91 (0.65, 1.26)
*p* trend				0.829				0.558
Nonfermented								
Q1	77	3.10	49,528.12	1	127	3.11	133,102.69	1
Q2	56	6.31	45,468.45	0.78 (0.51, 1.18)	150	6.45	140,376.58	1.07 (0.80, 1.44)
Q3	51	10.6	41,042.78	0.90 (0.59, 1.37)	165	10.5	144,277.5	1.17 (0.87, 1.56)
Q4	33	19.9	34,152.58	0.73 (0.45, 1.19)	166	20.5	150,554.79	1.23 (0.92, 1.65)
*p* trend				0.303				0.158

Note

HR: Adjusted for age, area, smoking status, number of cigarettes smoked per day, alcohol consumption, BMI, height, energy intake, physical activity, past history of diabetes, family history, history of breast tumor, received mammography, age at menarche, age at first birth, number of deliveries, hormone use, and breast feeding. The threshold for significance was *p* < 0.05.

Abbreviations: g, gram; mg, milligram; No, number; Q, quartile.

In subgroup analysis by BMI category, each category and individual food item showed no consistent association with breast cancer risk (data not shown).

## 4 DISCUSSION

This study found no association between soy food consumption and the risk of total breast cancer, regardless of fermentation status. In subgroup analyses, women with the highest intake of fermented soy foods had half the risk of nonlocalized breast cancer than women in the lowest intake category. No association was evident between fermented soy foods and breast cancer risk in premenopausal or postmenopausal women.

As expected, even though the number of cases analyzed was small and 95% CIs were wide, the highest quartile of fermented soy food intake showed a decreased risk of nonlocalized breast cancer. Unexpectedly, however, the same inverse association was not observed for overall or localized breast cancer. Some studies have reported that components of soybeans like isoflavone have anticancer activity.^34^ Fermented soy foods contain isoflavones as aglycone, which is absorbed more readily and faster than isoflavones occurring as glycoside in nonfermented soy foods.^36^, ^37^ Several laboratory studies reported that fermented soymilk exercised a larger antiproliferative effect than nonfermented soymilk, such as a reduction in breast cancer volume.^37^ One laboratory study showed that genistein substantially inhibited the proliferative ability of human breast cancer cell lines to a greater extent, particularly at high concentrations.^38^ Given these results, our study suggests the possibility that high fermented soy intake may prevent breast tumor growth rather than incidence. Interpreting this association requires care, however, because we can easily assume that localized cases are more likely to be detected through cancer screening. Those who consume more soy foods were more likely to undergo cancer screening than those who did not (Table 1). We also note that while fermented soy foods showed a significant trend, individual components of natto did not appear to contribute. In a laboratory study, amounts of free isoflavones increased with fermentation in miso, but hardly increased in natto.^39^ Longer duration necessary for fermentation for miso than natto may have contributed to the different effects on breast tumor development.^40^ Further studies, both epidemiological and experimental are necessary to clarify the association and its biological plausibility.

Most previous cohort studies showed no association between soy food intake and breast cancer risk. Our previous report^22^ showed an inverse association between breast cancer risk and the intake of miso soup and isoflavone, which is somewhat inconsistent with our present analysis. Possible reasons for this inconsistency include (1) only JPHC Cohort I was used in the previous study; (2) different age range (previous report aged 40–59 years); (3) shorter follow‐up time (9.6 years) in the previous analysis; (4) different questionnaires at different timings were used for dietary assessment, wherein the present analysis used FFQ at 5‐year follow‐up survey with eight soy food items while the previous analysis used baseline FFQ with two soy food items. In short, this study observed the longer‐term effect of soy foods with more detailed analysis, and a larger number of soy food items and outcomes. In addition, as mammography was introduced as governmental screening in Japan in 2000, the stage distribution of breast cancer cases in the previous report was different from the present analysis: the previous report consisted of 48% localized and 52% nonlocalized cases, while the current analysis included 61% localized and 39% nonlocalized cases. The higher proportion of advanced cases may have led to the clearer overall inverse association between soy food consumption and breast cancer risk in the previous report.

The effect of soy foods by menopausal status is also inconsistent among previous studies. One study^19^ found no association between risk of breast cancer and high intake of tofu or miso soup regardless of menopausal status. Our previous report^22^ found a stronger inverse association between soy food/isoflavone intake and breast cancer in postmenopausal women compared to premenopausal women whereas our study found no association in either premenopausal or postmenopausal women. The larger sample size and longer follow‐up time in this study may have led to more stable findings.

Several limitations of this study should be acknowledged. First, there could be a measurement error in assessment of the dietary data. The FFQ used in this study was not originally designed to test the effect of soy foods alone and other soy products such as soy sauce were not included in this analysis. There also may have been changes in dietary habits during the study period. However, the FFQ is a validated instrument for measuring dietary intake and can be used to rank individuals according to intake.^41^ It also reasonably reflects long‐term dietary intake.^31^ Second, there is a possibility of residual confounding by other dietary factors, although epidemiological studies have not confirmed any dietary items except alcohol as risk factors of breast cancer.^2^ In a previous study from the same cohort, a westernized dietary pattern indicated an association with an increased breast cancer risk.^3^ Fermented soy foods are commonly served in Japanese style dining, so our results may reveal the effect of Japanese eating habits. Third, there is a possibility of residual confounding due to health‐conscious behavior, which may also affect dietary pattern. As described above, subjects who consumed a relatively high amount of soy foods may be more health conscious and may undergo cancer screening more frequently than nonconsumers. Localized breast cancer is known to be substantially overdiagnosed in screening mammography.^42^ Unlike in the cases of advanced cancer or mortality, some bias or confounding in detecting localized breast cancer may be present. We adjusted for history of mammography in the analyses, but the questionnaire asked only whether subjects received a mammogram in the past year and was therefore not able to acquire the effect of health‐conscious behavior. Fourth, we could not identify menopausal status at the time breast cancer was diagnosed. Because hormone levels differ between premenopausal and postmenopausal women, fermented soy foods may affect the two groups differently. Fifth, we could not analyze by hormone receptor status because of the lack of data (missing in more than 60% of cases).

Despite these limitations, to our knowledge this is the first epidemiological study to examine the association between soy foods, with division by fermentation and the risk of breast cancer. Because Japanese consume a relatively high amount of soy foods, this study was also characterized by a wide range of soy intake. The study design, collecting exposure data before diagnosis, made recall and selection bias minimal. A very small number of participants were lost to follow‐up in this cohort (0.7%), and the high response rate to this study (83.5%) was also a major strength in minimizing selection bias. Breast cancer incidence was confirmed by the cancer registry not self‐report, reducing the potential for misclassification of the outcome. The participants of this study were enrolled from the general population and the incidence of breast cancer identified in this study did not differ from the national data of Japan. Therefore, the results of this study may be generalized to the general Japanese population, or to populations with a similar background.

In conclusion, we found an increase in fermented soy food intake was associated with a decrease in nonlocalized breast cancer risk. The reason why fermented soy foods would have an association with nonlocalized breast cancer but not localized breast cancer is yet to be clarified. Our findings warrant the need to consider the effects of fermented soy and total soy in the etiology of breast cancer and its prevention separately.

## CONFLICT OF INTEREST

All authors declare no conflicts of interest.

## AUTHOR CONTRIBUTION

M. Inoue, NS and ST designed and conducted research; RS analyzed data; RS, ES, SKA, and M. Inoue wrote the paper; NS, JI, RT, TS, TY, AG, M. Iwasaki, and ST made substantial contributions to strengthening the Subjects and Methods and Discussion sections. M. Inoue holds primary responsibility for final content. All authors read and approved the final manuscript.

## Data Availability

The data that support the findings of this study are available from the corresponding author upon reasonable request.

## References

[1] Bray F , Ferlay J , Soerjomataram I , Siegel RL , Torre LA , Jemal A . Global cancer statistics 2018: GLOBOCAN estimates of incidence and mortality worldwide for 36 cancers in 185 countries. CA Cancer J Clin. 2018;68(6):394‐424. 30207593 10.3322/caac.21492

[2] Hori M , Matsuda T , Shibata A , Katanoda K , Sobue T , Nishimoto H . Cancer incidence and incidence rates in Japan in 2009: a study of 32 population‐based cancer registries for the Monitoring of Cancer Incidence in Japan (MCIJ) project. Japanese journal of clinical oncology. 2015;45(9):884–91. 26142437 10.1093/jjco/hyv088

[3] Diet, Nutrition, Physical Activity and Cancer: A Global Perspective. Continuous Update Project Expert Report 2018. The World Cancer Research Fund/American Institute for Cancer Research; 2018.

[4] Shin S , Saito E , Inoue M , et al. Dietary pattern and breast cancer risk in Japanese women: the Japan Public Health Center‐based Prospective Study (JPHC Study). Br J Nutr. 2016;115(10):1769‐1779. 26997498 10.1017/S0007114516000684

[5] Yoshiike N , Matsumura Y . National nutrition survey in Japan. J Epidemiol. 1995;6:S189‐S200. 10.2188/jea.6.3sup_189 8800293

[6] Koba S , Tanaka H . Physical activity in the Japan population: association with blood lipid levels and effects in reducing cardiovascular and all‐cause mortality. J Atheroscler Thromb. 2011;18:833‐845. 21946534 10.5551/jat.8094

[7] Nagata C , Hu YH , Shimizu H . Effects of menstrual and reproductive factors on the risk of breast cancer: meta‐analysis of the case‐control studies in Japan. Jpn J Cancer Res. 1995;86(10):910‐915. 7493908 10.1111/j.1349-7006.1995.tb03000.x PMC5920604

[8] Messina M . Soy intake and cancer risk: a review of the in vitro and in vivo data. Nutr Cancer. 1994;21:113‐131. 8058523 10.1080/01635589409514310

[9] Tj K , Pn A , Gk R , et al. Steroid hormone measurements from different types of assays in relation to body mass index and breast cancer risk in postmenopausal women: reanalysis of eighteen prospective studies. Steroids. 2015;99:49‐55. 25304359 10.1016/j.steroids.2014.09.001 PMC4502556

[10] Zhang X , Tworoger SS , Eliassen AH , Hankinson SE . Postmenopausal plasma sex hormone levels and breast cancer risk over 20 years of follow‐up. Breast Cancer Res Treat. 2013;137(3):883‐892. 23283524 10.1007/s10549-012-2391-z PMC3582409

[11] Adlercreutz H , Goldin B , Gorbach S , et al. Soybean phytoestrogen intake and cancer risk. J Nutr. 1995;125:S757‐S770. 10.1093/jn/125.3_Suppl.757S 7884562

[12] Kurzer M , Xu X . Dietary phytoestrogens. Annu Rev Nutr. 1997;17:353‐381. 9240932 10.1146/annurev.nutr.17.1.353

[13] Trock BJ , Hilakivi‐Clarke L , Clarke R . Meta‐analysis of soy intake and breast cancer risk. J Natl Cancer Inst. 2006;98(7):459‐471. 16595782 10.1093/jnci/djj102

[14] Ying‐Chao W , Dong Z , Jin‐Jie S , Zhi‐Kang Z , Zhong‐Li M . Meta‐analysis of studies on breast cancer risk and diet in Chinese women. Int J Clin Exp Med. 2015;8(1):73‐85. 25784976 PMC4358431

[15] Greenstein J , Kushi L , Zheng W , Fee R , Campbell D , Sellers T . Risk of breast cancer associated with intake of specific foods and food groups. Am J Epidemiol. 1996;143:S36.

[16] Lital K‐B , TvDS Y , Diederick EG , Petra HP . Dietary phytoestrogens and breast cancer risk. Am J Clin Nutr. 2004;79(2):282‐288. 14749235 10.1093/ajcn/79.2.282

[17] Nishio K , Niwa Y , Toyoshima H , et al. Consumption of soy foods and the risk of breast cancer: findings from the Japan Collaborative Cohort (JACC) Study. Cancer Causes Control. 2007;18(8):801‐808. 17619154 10.1007/s10552-007-9023-7

[18] Horn‐Ross PL , Hoggatt KJ , West DW , et al. Recent diet and breast cancer risk: the California Teachers Study (USA). Cancer Causes Control. 2002;13:407‐415. 12146845 10.1023/a:1015786030864

[19] Hirayama T . Life‐style and cancer: from epidemiological evidence to public behavior change to mortality reduction of target cancers. J Natl Cancer Inst Monogr. 1992;12:65‐74. 1616813

[20] Key T , Sharp G , Appleby P , et al. Soya foods and breast cancer risk: a prospective study in Hiroshima and Nagasaki, Japan. Br J Cancer. 1999;81(7):1248‐1256. 10584890 10.1038/sj.bjc.6690837 PMC2374337

[21] Wu AH , Yu MC , Tseng CC , Pike MC . Epidemiology of soy exposures and breast cancer risk. Br J Cancer. 2008;98(1):9‐14. 18182974 10.1038/sj.bjc.6604145 PMC2359677

[22] Toi M , Hirota S , Tomotaki A , et al. Probiotic beverage with soy isoflavone consumption for breast cancer prevention: a case‐control study. Curr Nutr Food Sci. 2013;9:194‐200. 23966890 10.2174/15734013113099990001 PMC3744907

[23] Yamamoto S , Sobue T , Kobayashi M , Sasaki S , Tsugane S . Soy, isoflavones, and breast cancer risk in Japan. J Natl Cancer Inst. 2003;95(12):906‐913. 12813174 10.1093/jnci/95.12.906

[24] Tsugane S , Sawada N . The JPHC study: design and some findings on the typical Japanese diet. Jpn J Clin Oncol. 2014;44(9):777‐782. 25104790 10.1093/jjco/hyu096

[25] Cancer Registry and Statistics . Cancer Information Service, National Cancer Center, Japan . Vital Statistics Japan (Ministry of Health, Labour and Welfare).

[26] Sasaki S , Kobayashi M , Ishihara J , Tsugane S . Self‐administered food frequency questionnaire used in the 5‐year follow‐up survey of the JPHC Study: questionnaire structure, computation algorithms, and area‐based mean intake. J Epidemiol. 2003;13(1):S13‐S22. 12701629 10.2188/jea.13.1sup_13 PMC9767697

[27] Yamamoto S , Sobue T , Sasaki S , et al. Validity and reproducibility of a self‐administered food‐frequency questionnaire to assess isoflavone intake in a Japanese population in comparison with dietary records and blood and urine isoflavones. J Nutr. 2001;131:2741‐2747. 11584098 10.1093/jn/131.10.2741

[28] Arai Y , Watanabe S , Kimira M , Shimoi K , Mochizuki R , Kinae N . Dietary intakes of flavonols, flavones and isoflavones by Japanese women and the inverse correlation between quercetin intake and plasma LDL cholesterol concentration. J Nutr. 2000;130:2243‐2250. 10958819 10.1093/jn/130.9.2243

[29] Kimira M , Arai Y , Shimoi K , Watanabe S . Japanese intake of flavonoids and isoflavonoids from foods. J Epidemiol. 1998;8:168‐175. 9782673 10.2188/jea.8.168

[30] Nanri A , Shimazu T , Ishihara J , et al. Reproducibility and validity of dietary patterns assessed by a food frequency questionnaire used in the 5‐year follow‐up survey of the Japan Public Health Center‐Based Prospective Study. J Epidemiol. 2012;22(3):205‐215. 22343330 10.2188/jea.JE20110087 PMC3798621

[31] Sasaki S , Ishihara J , Tsugane S . Reproducibility of a self‐administered food frequency questionnaire used in the 5‐year follow‐up survey of the JPHC Study Cohort I to assess food and nutrient intake. J Epidemiol. 2003;13(1 Sup):S115‐S124. 12701639 10.2188/jea.13.1sup_115 PMC9767696

[32] Ishihara J , Sobue T , Yamamoto S , et al. Validity and reproducibility of a self‐administered food frequency questionnaire in the JPHC Study Cohort Ⅱ: study design, participant profile and results in comparison with Cohort Ⅰ. J Epidemiol. 2003;13(1 Supplement):S134‐S147. 12701641 10.2188/jea.13.1sup_134 PMC9767691

[33] Willett W , Howe G , Kushi L . Adjustment for total energy intake in epidemiologic studies. Am J Clin Nutr. 1997;65:S1220‐S1228. 10.1093/ajcn/65.4.1220S 9094926

[34] Wada K , Nagata C , Tamakoshi A , et al. Body mass index and breast cancer risk in Japan: a pooled analysis of eight population‐based cohort studies. Ann Oncol. 2014;25(2):519‐524. 24412821 10.1093/annonc/mdt542

[35] Messina M . Modern applications for an ancient bean: soybeans and the prevention and treatment of chronic disease. J Nutr. 1995;125:S567‐S569. 10.1093/jn/125.3_Suppl.567S 7884533

[36] Huei‐ju W , Patricia AM . Isoflavone content in commercial soybean foods. J Agric Food Chem. 1994;42:1666‐1673.

[37] Izumi T , Piskula MK , Osawa S , et al. Soy isoflavone aglycones are absorbed faster and in higher amounts than their glucosides in humans. J Nutr. 2000;130:1695‐1699. 10867038 10.1093/jn/130.7.1695

[38] Lai LR , Hsieh SC , Huang HY , Chou CC . Effect of lactic fermentation on the total phenolic, saponin and phytic acid contents as well as anti‐colon cancer cell proliferation activity of soymilk. J Biosci Bioeng. 2013;115(5):552‐556. 23290992 10.1016/j.jbiosc.2012.11.022

[39] Jiang H , Fan J , Cheng L , Hu P , Liu R . The anticancer activity of genistein is increased in estrogen receptor beta 1‐positive breast cancer cells. Onco Targets Ther. 2018;11:8153‐8163. 30532556 10.2147/OTT.S182239 PMC6241715

[40] Esaki H , Onozaki H , Osawa T . Antioxidative Activity of Fermented Soybean Products. Food Phytochemicals for Cancer Prevention I ACS Symposium Series. 546. American Chemical Society; 1993: p. 353‐360.

[41] Watanabe H . Beneficial biological effects of miso with reference to radiation injury, cancer and hypertension. J Toxicol Pathol. 2013;26(2):91‐103. 23914051 10.1293/tox.26.91 PMC3695331

[42] Sasaki S , Kobayashi M , Tsugane S . Validity of a self‐administered food frequency questionnaire used in the 5‐year follow‐up survey of the JPHC Study Cohort I: comparison with dietary records for food groups. J Epidemiol. 2003;13(1):S57‐S63. 12701632 10.2188/jea.13.1sup_57 PMC9767694

[43] Bleyer A , Welch HG . Effect of three decades of screening mammography on breast‐cancer incidence. N Engl J Med. 2012;367(21):1998‐2005. 23171096 10.1056/NEJMoa1206809

